# The diagnostic performance of musculoskeletal ultrasound in gout: A systematic review and meta-analysis

**DOI:** 10.1371/journal.pone.0199672

**Published:** 2018-07-06

**Authors:** Qingyu Zhang, Fuqiang Gao, Wei Sun, Jinhui Ma, Liming Cheng, Zirong Li

**Affiliations:** 1 Graduate School of Peking Union Medical College, China-Japan Friendship Institute of Clinical Medicine, Chaoyang District, Beijing, China; 2 Centre for Osteonecrosis and Joint-preserving & Reconstruction, Orthopaedic Department, China-Japan Friendship Hospital, Chaoyang District, Beijing, China; 3 Graduate School of Peking Union Medical, Centre for Osteonecrosis and Joint-preserving & Reconstruction, Orthopaedic Department, China-Japan Friendship Hospital, Chaoyang District, Beijing, China; 4 Beijing Key Laboratory of Arthritic and Rheumatic Diseases, China-Japan Friendship Institute of Clinical Medicine, Beijing, China; Drexel University, UNITED STATES

## Abstract

**Background:**

Musculoskeletal ultrasound is widely used in diagnosing gout, but its accuracy is debatable. We conducted a systematic review and meta-analysis to quantitatively evaluate the value of ultrasound in the diagnosis of gout.

**Methods:**

We systematically searched for publications using Cochrane Library, PubMed/Medline and Embase and manually screened the references of eligible articles for additional relevant publications. Studies were included in this systematic review if they assessed the diagnostic accuracy of ultrasound in gout compared to that of the gold standard, demonstration of monosodium urate crystals in joint fluid or tophi. We then conducted quantitative analyses by extracting data from each study and calculating the pooled sensitivity, specificity, positive likelihood ratio (PLR), negative likelihood ratio (NLR) and diagnostic odds ratio (DOR). The summary receiver operating characteristic curves (sROCs) were constructed to obtain the Q*-index and the area under the curve (AUC).

**Results:**

Thirteen studies were included in this meta-analysis. The diagnostic performances of three distinctive ultrasonographic features of gout, double contour sign (DCS), the presence of tophi and the snowstorm sign, were evaluated. For person-based evaluations, the pooled sensitivity, specificity, DOR, AUC and Q* were as follows: for the DCS, 66% (95% confidence interval (CI) 62%-69%), 92% (95% CI 90%-94%), 25.91 (95% CI 11.80–56.89), 0.8163 and 0.7503, respectively; for the presence of tophi, 56% (95% CI 52%-60%), 94% (95% CI 92%-96%), 21.11 (95% CI 7.84–56.89), 0.8928 and 0.8236, respectively; for the snowstorm sign, 31% (95% CI 27%-36%), 91% (95% CI 88%-93%), 4.54(95% CI 3.13–6.58), 0.5946 and 0.5712, respectively; and for simultaneous consideration of these ultrasonographic features, 80% (95% CI 76%-83%), 83% (95% CI 79%-86%), 19.03 (95% CI 13.97–25.93), 0.889 and 0.8197, respectively. For the joint-/location-based evaluations, the pooled sensitivity, specificity, DOR, AUC and Q* were as follows: for the DCS, 75% (95% CI 68%-80%), 65% (95% CI 59%-70%), 16.90 (95% CI 5.10–56.03), 0.871 and 0.8014, respectively; and for the presence of tophi, 48% (95% CI 40%-57%), 96% (95% CI 91%-99%), 30.20 (95% CI 9.23–98.87), 0.8776 and 0.8081, respectively.

**Conclusions:**

In this meta-analysis, relatively high specificity but modest or low sensitivity were demonstrated in the diagnosis of gout using each of the three ultrasonographic features for person-based evaluations. Simultaneous consideration of these ultrasound findings may improve the diagnostic sensitivity. However, the double contour sign alone is weak in the differentiation of gout and non-gout for joint-/location-based evaluations. Further well-designed studies are still needed to support the current findings.

## Introduction

Gout is a disorder characterized by disturbances in purine metabolism and urate excretion, and it is the most common type of inflammatory arthritis [1]. Currently, the demonstration of monosodium urate (MSU) crystals in aspirate from synovial fluid or tophi serves as the gold standard for diagnosing gout [1]. However, arthrocentesis is an invasive test and is not always practical for all patients. As a result, treatment of gout is often initiated based on clinical evidence only [2, 3]. An accurate and convenient modality for the diagnosis of gout is imperative.

Musculoskeletal ultrasounds (US) can detect crystal deposition in various anatomical areas. It can be used not only for guiding aspiration but also for diagnosing gout, and was therefore incorporated into the gout diagnostic criteria of the American College of Rheumatology and the European League against Rheumatism [4]. The three features including the double contour sign (DCS), presence of tophi and the snowstorm sign are suggestive of urate deposition on US and have been widely investigated, while other sonographic signs such as gouty-like bone erosions, synovitis and joint effusion are believed to be non-specific for gout [5–8]. According to the consensus of the Outcome Measures in Rheumatology (OMERACT) Ultrasound Gout Task Force group, the DCS is the hyperechoic irregular enhancement (urate crystals deposits) of the articular surface of the hyaline cartilage; the floating hyperechoic crystals within the joint space have the appearance of a snowstorm; tophi appear as hyperechoic to hypoechoic, heterogenous material with poorly defined borders [9]. In real life clinical practice, sonographers screen gout by simultaneously considering these features [6–8].

A series of studies have investigated the diagnostic accuracy of ultrasound for gout; however, various methodological differences exist and their results are controversial. Several systematic reviews qualitatively evaluated these studies but did not calculate the pooled diagnostic accuracy [5, 6, 10, 11]. A meta-analysis published in 2015 by Ogdie et al further clarified this issue, but the following should be considered in interpreting its results [7]: first, the meta-analysis only included 7 studies, among which 5 provided data about the DCS, 5 provided data about tophi and data regarding the snowstorm sign were unavailable for quantitative pooling. More importantly, the investigators did not differentiate data of person-based evaluations from joint-/location-based evaluations. In person-based evaluations, the ultrasound is performed on multiple joints in a single person for diagnostic purpose while in joint-/location-based evaluations the ultrasound is performed only for symptomatic joints/locations. The data from these two types of evaluations have different implications and should not be combined. Furthermore, subgroup analyses by study design (case-control or cross-sectional) were not performed. In this updated meta-analysis, we included newly published studies, evaluated more outcome measures, and analyzed data from person-based and joint-/location-based evaluations separately to further clarify the diagnostic accuracy of ultrasound for gout and provide more reliable evidence for clinicians.

## Methods

This study was conducted according to the Preferred Reporting Items for Systematic Reviews and Meta-Analyses (PRISMA) Statement [12]. All data analyzed were extracted from published studies. Therefore, no ethical approval or written informed consent was required. The database search, literature selection and assessment, and data extraction were conducted by two investigators (Zhang QY and Gao FQ) independently, and any disagreement was resolved by discussion and consensus.

### Search strategy

The literature search was conducted using combinations of the following keywords: 1) “ultrasound” OR “ultrasonic” OR “ultrasonography” OR “sonography” OR “echography” OR “US” AND 2) “gout” OR “urate” OR “uric acid”. The search strategy was conducted in Medline/PubMed (from 1966 to April 2017), Embase (from 1975 to April 2017) and Cochrane Library (no time restriction). No language limitation was imposed. The references of relevant articles were also hand searched to retrieve additional eligible articles.

### Selection criteria and literature assessment

Study eligibility for this systematic review was based on the following inclusion criteria: a) those assessing the performance of musculoskeletal ultrasound in the diagnosis of gout and b) the diagnosis of gout was confirmed by detection of monosodium urate crystals. For studies analyzing both gout and non-gout participants, if the numbers of true-positive (TP), false-positive (FP), false-negative (FN) and true-negative (TN) cases were reported, they were included for quantitative analysis. If any articles included overlapping data, the most comprehensive or recent article was selected.

Exclusion criteria included the following: a) in vitro or animal studies; b) those including patients with asymptomatic hyperuricemia; and c) unoriginal research (reviews, editorials, meta-analyses, letters and comments).

Articles obviously unrelated to the objectives of this study were excluded by screening titles and abstracts. Full texts of remaining articles were then reviewed carefully.

### Data extraction and methodological quality assessment

Data extracted from eligible studies included publication year, study design, inclusion and exclusion criteria, parameters of the sonographic method used, qualification of sonographers, number of examined joints and the diagnostic method that is considered the gold standard. These data were recorded in a predesigned table while demographic information of involved participants was recorded in another. Reported values for TP, FP, FN and TN for each study were collected for quantitative pooling. The methodological quality of the studies was assessed by using the QUADAS-1 (Quality Assessment of Diagnostic Accuracy Studeis-1) tool, which consists 14 questions [13]. One point was given for every QUADAS-1 criterion met by the study while unmet or unclear items were given a score of zero. The final results of the methodological quality assessment were summarized with Revman version 5.3.5 (The Cochrane Collaboration, Copenhagen, Denmark).

### Statistical analysis

The pooled analyses were performed using Metadisc version 1.4 (Unit of Clinical Biostatistics team of the Ramón y Cajal Hospital, Madrid, Spain). Pooled sensitivity, specificity, positive likelihood ratio (PLR), negative likelihood ratio (NLR), diagnostic odds ratio (DOR), and their 95% confidence intervals (CIs) of ultrasound for the diagnosis of gout were obtained using the random-effects model. A pooled DOR ranges from zero to infinity and a higher pooled DOR represents better accuracy. The summary receiver operating characteristic curve (sROC) was created to obtain the area under the curve (AUC) and Q*-index, which comprehensively reflect diagnostic accuracy. On the sROC, each point represents a single study with the x-axis representing sensitivity and the y-axis representing specificity. Q* is the point on the sROC where the sensitivity equals the specificity. Data from person-based and joint-/location-based evaluations were pooled separately. In the person-based evaluations, several joints or locations of one subject were tested, and by considering the ultrasound abnormalities of these joints/locations, the diagnosis was made. In the joint-/location-based data evaluations, only the symptomatic joints/locations were evaluated using ultrasound rather than the whole subject. The Deek’s test was performed to detect publication bias. A p-value of less than 0.1 indicated a significant publication bias.

## Results

### Study selection

**Fig 1:**
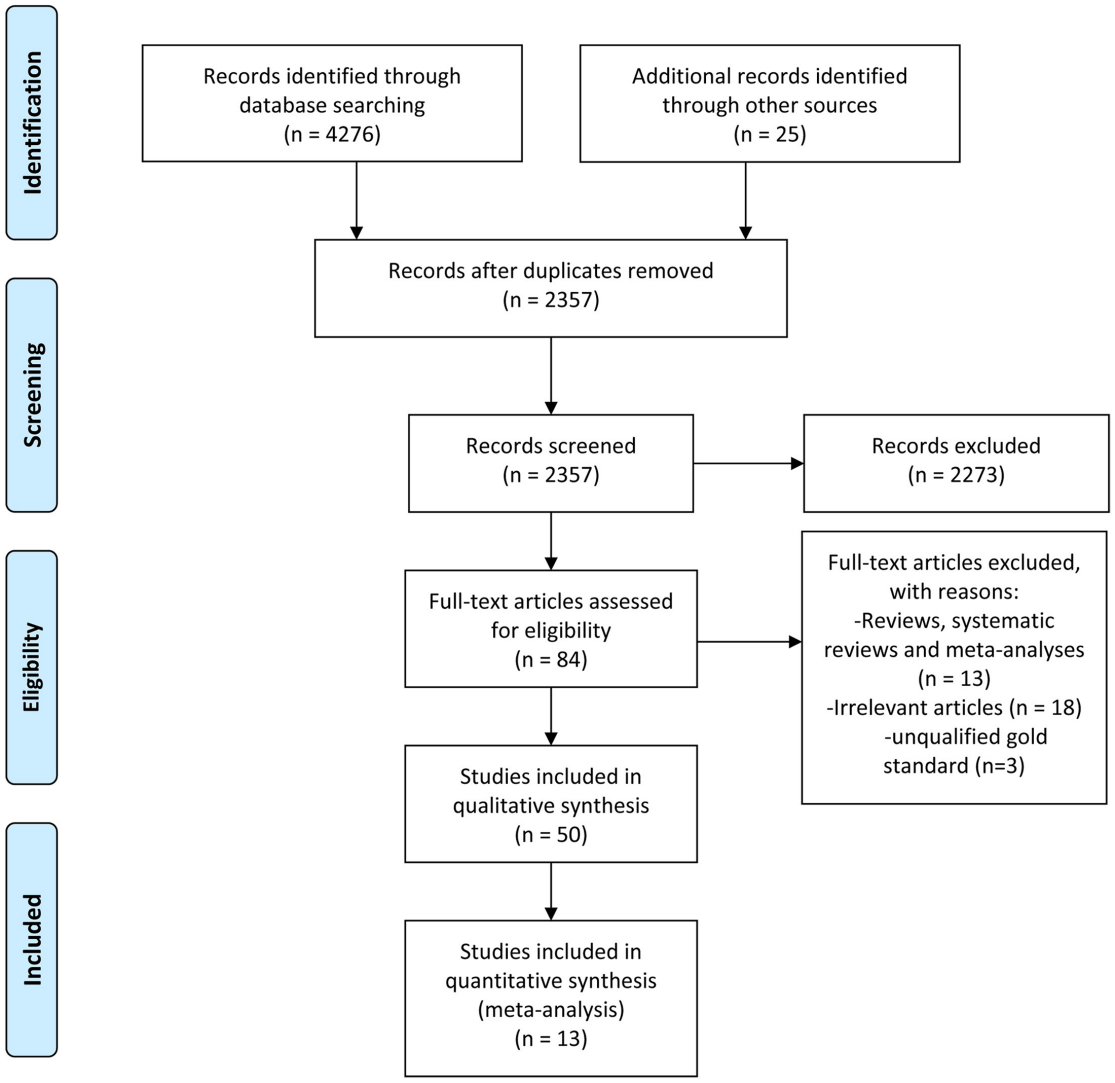
A flow chart summarizing the study selection process for this meta-analysis.

According to the procedures outlined in the PRISMA statement, we identified 13 studies [14–26] for quantitative analysis from the three electronic databases. The selection process and the reasons for study exclusion in each step are depicted in Fig 1.

### The characteristics of the studies and involved participants

**Fig 2:**
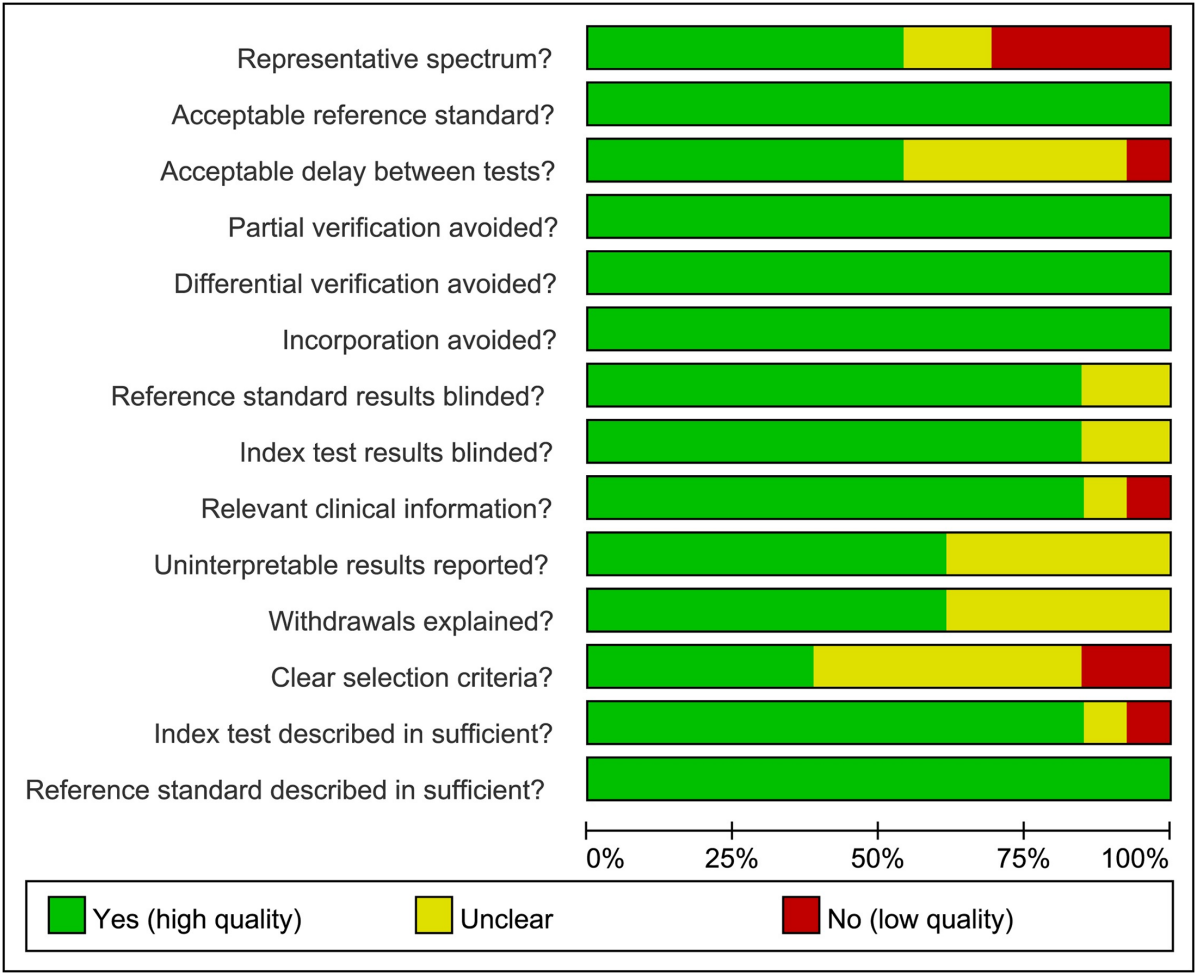
Results of the methodological quality evaluations using the QUADAS-1 tool. Green indicates that the criterion is satisfied. Yellow means that it is unclear whether the criterion is satisfied or not. Red indicates that the study did not meet the criterion.

Among the 13 studies included in the meta-analysis, 12 [14–18, 20–26] were published in English and one [19] was published in Chinese; 12 [14–22, 24–26] were published as manuscripts and one [23] was a conference abstract; 6 [15, 19, 22, 24–26] were case-control studies and 7 [14, 16–18, 20, 21, 23] were cross-sectional studies. 6 [16, 17, 21, 23, 25, 26] studies presented data from joint-/location-based evaluations while 7 [14, 15, 18–20, 22, 24] studies provided data from person-based evaluations. Among 7 studies conducting person-based evaluations, 1 [14] only carried out US on symptomatic joints/locations, 2 [18, 20] on symptomatic joints as well as several predetermined joints, and 4 [15, 19, 22, 24] on same joints/locations in different participants irrespective of their clinical manifestations. 11 [14–22, 24, 25] studies specifically stated that the results of the diagnostic gold standard were unknown to the sonographers and the other 2 [23, 26] studies did not specify this fact. The details of the 13 included studies and involved participants are presented in Tables 1 and 2, respectively. The results of the methodological quality evaluation are summarized in Fig 2. The main methodological flaws among the included articles were the lack of information about uninterpretable results, withdrawals, selection criteria and sonographic parameters, and other clinical information.

**Table 1:** Characteristics of included studies.

Study	Study design	Inclusion interval	Probe specifications	Qualifications of sonographers	Joints examined
Ogdie, 2017 [14]	multicenter cross-sectional study	January 2013-April 2014	NR	all ultrasonographers had prior US training	one or more clinically affected joints
Das, 2017 [15]	case-control study	June,2011-December,2012	12–18 MHz linear probe	a rheumatologist trained in musculoskeletal US with experience for more than 4 years	both the first MTP joints and both of the knee joints
Elsaman, 2016 [16]	prospective single-center cross-sectional study	NR	8–12 MHz linear probe	NR	131 joints with arthritis (33 MTP 1 joints and 98 knee joints)
Loffler, 2015 [17]	retrospective cross-sectional study	NR	7–14 MHz linear probe	sonographers trained in joint sonography and certified by the standards of the DEGUM with at least 2 years of experience in joint sonography	225 acutely inflamed joints including knee, ankle, wrist, finger, MTP 1, shoulder, elbow joints
Zufferey, 2015 [18]	prospective single-center cross-sectional study	October,2012-May 2014	9–18 MHz	one rheumatologist with over 15 years of experience and the other with 2 years of experience	the symptomatic joints as well as both knee, ankle and MTP 1 joints
Leng, 2014 [19]	single-center case-control study	January 2009-December 2012	8–12 MHz	two sonographers with more than 6 years of experience of musculoskeletal US	knee, ankle, wrist, finger, MTP, MCP, shoulder, elbow joints
Lamers-Karnebeek, 2014 [20]	prospective single-center cross-sectional study	NR	10–18 MHz and 13–14 MHz	two rheumatologists and two trainees	the joint with arthritis, the contralateral side and two other joints bilaterally
Gruber, 2014 [21]	prospective single-center cross-sectional study	March 2010-April 2012	15 or 18 MHz	two US examiners with 8 and 10 years of experience in musculoskeletal US	37 suspected joints the hands, wrists, feet, ankles, knees and elbows
Naredo, 2014 [22]	multicenter case-control study	NR	12–14 MHz liner probe	a rheumatologist highly experienced in musculoskeletal US at each center (i.e., 10–15 years of experience)	1 joint (radiocarpal joint), 2 tendons (patellar and triceps) and 3 articular cartilages (first MTP, talar and second metacarpal/femoral)
Bergner, 2013 [23]	cross-sectional study	NR	NR	NR	113 symptomatic joints including knee (n = 74), small finger or toe (n = 10), elbow (n = 5), ankle (n = 12), shoulders (n = 6) and wrists (n = 6)
Ottaviani, 2012 [24]	single-center case-control study	November 2008-October 2010	7–15 MHz multilinear probe	2 rheumatologists trained in musculoskeletal US	The first and second MTP joints, both knees and the second and third MCP joints
Thiele, 2007 [25]	retrospective single-center case-control study	November 2003-December 2004	5–10 MHz linear probe	a rheumatologist certified in musculoskeletal US and a second rheumatologist with a short instruction period	70 symptomatic joints including MCP, MTP 1, humero-radial, knee, shoulder, elbow joints
Nalbant, 2003 [26]	prospective case-control study	May 2001-October 2001	10–15 MHz linear probe	2 rheumatologists trained in musculoskeletal US and experienced in using portable US	20 nodules located on the finger, elbow, wrist and MTP joints

DEGUM, the German Society of US in Medicine; MTP, metatarsophalangeal; MCP, metacarpophalangeal; MSU, monosodium urate; US, ultrasound; NR, not reported

**Table 2:** Characteristics of included participants.

Study	Number of included participants	Gout/control participants	Number of involved joints or locations	Joints or locations with positive/negative PLM result for MSU	Mean age (gout/control) (years)	Male: female (gout/control)	Duration of gout	SUA level (Mean±SD)	Conditions of control participants
Ogdie, 2017 [14]	824	416/408	1191	NA	60.2±14.6/59.5±16.0	363:53/222:186	NR	0.466±0.138 mmol/L	CPPD, clinically suspected gout, OA, RA, spondyloarthropathies, undifferentiated inflammatory arthritis, septic arthritis, systemic lupus erythematosus, and other
Das, 2017 [15]	92	62/30	NR	NA	49.13±9.1/47.6±10.6	60:2/29:1	58.48±36.4 months (range 12–144 months)	7.69±1.6 mg/dl	RA, OA and healthy individuals
Elsaman, 2016 [16]	100	47/53	131	71/60	55.06±6.42/NR	30:17/25:28	NR	NR	CPPD, non-CRA
Loffler, 2015 [17]	NR	NR	216	74/142	69±12/NR	NR	NR	NR	CPPD, non-CRA such as RA, PSA, septic arthritis, OA, hemarthrosis, reactive joint effusion after overexertion and others
Zufferey, 2015 [18]	109	60/49	NR	NA	65±12/NR	55:5/NR	<10 days	NR	CPPD, non-CRA
Leng, 2014 [19]	68	32/36	NR	NA	56.7±15.2/50.8±17.9	30:2/10:26	9.0±4.5 years	0.614±0.119 mmol/L	CPPD, OA, RA, HADD, synovial chondroma
Lamers-Karnebeek, 2014 [20]	54	26/28	NR	NA	63.5/55.0	25:1/13:15	new onset	0.52 mmol/L	CPPD, reactive arthritis, poly-arthritis eci, ankle arthritis in Loffgren triad, OA, discrete synovitis in PMR, PSA, undifferentiated arthritis
Gruber, 2014 [21]	21	NA	37	12/2	55.2±13.6	NA	acute and chronic	NR	NA
Naredo, 2014 [22]	133	91/42	NR	NA	56.4±11.5/56.6±13.5	NR	87.4(1–480) months	9±1.9 mg/dl	RA, spondyloarthritis and healthy subjects
Bergner, 2013 [23]	113	39/74	113	39/74	NR	NR	NR	NR	CPPD, non-CRA
Ottaviani, 2012 [24]	103	53/50	NR	NA	59.7±15.8/59.5±15.3	49:4/40:10	9.2±10.7 years	0.657±0.145 mmol/L	PSA, RA, CPPD, OA
Thiele, 2007 [25]	46	23/23	70	37/33	59.9±16.1/NR	17:6/8:15	>6 months (except one)	NR	CPPD, retrocalcaneal bursitis, inflammatory oligoarthritis, sarcoidosis, RA, lateral epicondylitis, OA, FMS, PSA, muscle fiber tear, bursitis, tendinitis
Nalbant, 2003 [26]	23	10/13	40	20/20	61.3±8.45/54.54±7.01	NR	10.7±3.13 years	NR	RA

PLM, polarized light microscopy; CPPD, chondrocalcinosis; HADD, hydroxyapatite deposition disease; non-CRA, non-crystal-related inflammatory joint disease; RA, rheumatoid arthritis; PSA, psoriatic arthritis; OA, osteoarthritis; FMS, fibromyalgia; PMR, polymyalgia rheumatica; NA, not available; NR, not reported

### Diagnostic accuracy of ultrasound

In the person-based evaluations, the pooled sensitivities and specificities for the diagnosis of gout were as follows: for the DCS, 0.66 (95% CI 0.62–0.69) and 0.92 (95% CI 0.89–0.94), respectively; for presence of tophi, 0.56 (95% CI 0.52–0.60) and 0.94 (95% CI 0.92–0.96), respectively; for the snowstorm sign, 0.31 (95% CI 0.27–0.36) and 0.91 (95% CI 0.88–0.93), respectively; and for simultaneous consideration of ultrasonographic features, 0.80 (95% CI 0.76–0.83) and 0.83 (95% CI 0.79–0.86), respectively (Table 3).

**Table 3:** Diagnostic accuracy of ultrasound for gout.

Ultrasonic feature	Evaluation method	Study	TP	FP	FN	TN	Pooled sensitivity	Pooled specificity	Pooled PLR	Pooled NLR	Pooled DOR	AUC	Q-index	P value of Deek’s plot
DCS	Patient	Ogdie, 2017 [14]	249	35	165	373	0.66 (0.62–0.69)	0.92 (0.89–0.94)	7.36 (3.81–14.24)	0.31 (0.24–0.41)	25.91 (11.80–56.89)	0.8163	0.7503	0.19
		Das, 2017 [15]	43	0	19	30								
		Leng, 2014 [19]	26	1	6	35								
		Lamers-Karnebeek, 2014 [20]	20	7	6	21								
		Naredo, 2014 [22]	68	7	23	35								
		Ottaviani, 2012 [24]	41	1	12	49								
	Joint/location	Elsaman, 2016 [16]	30	2	41	58	0.75 (0.68–0.80)	0.65 (0.59–0.70)	3.66 (1.36–9.82)	0.24 (0.07–0.79)	16.90 (5.10–56.03)	0.871	0.8014	0.363
		Loffler,2015 [17]	65	51	9	91								
		Bergner, 2013 [23]	36	21	3	53								
		Thiele, 2007 [25]	34	0	3	26								
Presence of tophi	Patient	Ogdie, 2017 [14]	189	21	222	387	0.56 (0.52–0.60)	0.94 (0.92–0.96)	7.25 (2.89–18.15)	0.38 (0.24–0.61)	21.11 (7.84–56.89)	0.8928	0.8236	0.491
		Das, 2017 [15]	41	0	21	30								
		Lamers-Karnebeek, 2014 [20]	5	2	21	26								
		Naredo, 2014 [22]	81	11	10	31								
		Ottaviani, 2012 [24]	42	0	11	50								
	Joint/location	Elsaman, 2016 [16]	20	0	51	60	0.48 (0.40–0.57)	0.96 (0.91–0.99)	14.05 (2.17–91.19)	0.40 (0.16–1.02)	30.20 (9.23–98.87)	0.8776	0.8081	0.909
		Thiele, 2007 [25]	27	0	10	26								
		Nalbant, 2003 [26]	15	4	5	24								
Snowstorm sign	Patient	Ogdie, 2017 [14]	125	37	287	370	0.31 (0.27–0.36)	0.91 (0.88–0.93)	3.40 (2.48–4.66)	0.76 (0.71–0.81)	4.54 (3.13–6.58)	0.5946	0.5712	0.625
		Leng, 2014 [19]	13	2	19	34								
		Lamers-Karnebeek, 2014 [20]	10	4	16	24								
Overall consideration	Patient	Ogdie, 2017 [14]	320	64	96	344	0.80 (0.76–0.83)	0.83 (0.79–0.86)	4.38 (3.51–5.45)	0.23 (0.17–0.31)	19.03 (13.97–25.93)	0.889	0.8197	0.228
		Zufferey, 2015 [18]	50	11	10	38								
		Lamers-Karnebeek, 2014 [20]	25	9	1	19								
		Naredo, 2013 [22]	77	7	14	35								

In the joint-/location-based evaluations, the pooled sensitivities and specificities for the diagnosis of gout were as follows: for DCS, 0.75 (95% CI 0.68–0.80) and 0.65 (95% CI 0.59–0.70), respectively, and for the presence of tophi, 0.48 (95% CI 0.40–0.57) and 0.96 (95% CI 0.91–0.99), respectively (Table 3).

Additionally, subgroup analyses were performed by study design.

Three [16, 17, 23] cross-sectional studies assessed the diagnostic value of the DCS on US in joint-/location-based evaluations; the pooled sensitivity and specificity were 0.71 (95% CI 0.64–0.78) and 0.62 (95% CI 0.56–0.67), respectively. Three [15, 18, 20] cross-sectional studies assessed the diagnostic value of simultaneous consideration of ultrasonographic features in person-based evaluations, the pooled sensitivity and specificity were 0.79 (95% CI 0.75–0.83) and 0.83 (95% CI 0.79–0.86), respectively (Table 4).

**Table 4:** Subgroup analyses of diagnostic accuracy of ultrasound for gout.

Study design	Ultrasonic feature	Evaluation method	Study	TP	FP	FN	TN	Pooled sensitivity	Pooled specificity	Pooled PLR	Pooled NLR	Pooled DOR	AUC	Q-index	P value of Deek’s plot
Cross-sectional study	DCS	joint/location	Elsaman, 2016 [16]	30	2	41	58	0.71 (0.64–0.78)	0.62 (0.56–0.67)	2.59 (1.12–5.98)	0.32 (0.10–1.04)	11.00 (5.34–22.68)	0.8549	0.7859	0.669
			Loffler, 2015 [17]	65	51	9	91								
			Bergner, 2013 [23]	36	53	3	21								
	Overall consideration	patient	Ogdie,2017 [15]	320	64	96	344	0.79 (0.75–0.83)	0.83 (0.79–0.86)	4.32 (3.45–5.41)	0.23 (0.16–0.35)	18.42 (13.31–25.48)	0.8869	0.8175	0.264
			Zufferey, 2015 [18]	50	11	10	38								
			Lamers-Karnebeek, 2014 [20]	25	9	1	19								
Case-control study	DCS	patient	Das, 2017 [15]	43	0	19	30	0.75 (0.69–0.80)	0.94 (0.89–0.97)	17.37 (3.59–84.01)	0.28 (0.22–0.35)	66.77 (13.58–328.22)	0.8159	0.7499	0.355
			Leng, 2014 [19]	26	1	6	35								
			Naredo, 2014 [22]	68	7	23	35								
			Ottaviani, 2012 [24]	41	1	12	49								
	Presence of tophus	patient	Das, 2017 [15]	41	0	21	30	0.80 (0.73–0.85)	0.91 (0.84–0.95)	18.57 (0.93–371.55)	0.23 (0.14–0.40)	71.51 (9.73–525.58)	0.9269	0.8614	0.747
			Naredo, 2014 [22]	81	11	10	31								
			Ottaviani, 2012 [24]	42	0	11	50								

Four [15, 19, 22, 24] case-control studies assessed the diagnostic value of the DCS on US in patient-based evaluations; the pooled sensitivity and specificity were 0.75 (95% CI 0.69–0.80) and 0.94 (95% CI 0.89–0.97), respectively. Three [15, 22, 24] case-control studies diagnosed gout by detecting tophi on US in person-based evaluations; the pooled sensitivity and specificity were 0.80 (95% CI 0.73–0.85) and 0.91 (95% CI 0.84–0.95), respectively (Table 4).

For each subgroup analyses, the reported rates for TP, FP, FN and TN, as well as pooled values for PLR, NLR, sDOR, AUC and Q* are listed in Tables 3 and 4. No significant publication bias was found using Deek’s test (all p-values > 0.1).

## Discussion

The utility of US in the diagnosis of gout is debatable, and arthrocentesis remains the gold standard to make a definitive diagnosis of gouty arthritis [1]. The variability in the performance of ultrasound in diagnosing gout reported by existing studies can be attributed to the following: the study design, the ultrasound equipment used, the experience of the sonographers, the sonography features used to diagnose gout, the method of ultrasound evaluation (joint-/location- or person-based), the number of joints tested, etc. In this systematic review and meta-analysis, we reviewed 13 studies, assessed the diagnostic performance of three ultrasound features, pooled data from person-based and joint-/location-based evaluations separately and performed subgroup analyses according to study design, which were not realized in a previous meta-analysis by Ogdie et al [7]. The current study aimed to clarify the value of musculoskeletal ultrasound in the diagnosis of gout.

Data from joint-/location-based evaluations and person-based evaluations should be analyzed separately. In joint-/location- based analysis, only symptomatic joints/locations were tested, and all diagnoses were confirmed by demonstration of MSU in synovial joint fluid or tophi. Patients with gout can have symptoms in other joints/locations that are due to other diseases or can be found to have MSU-negative under polarized light microscopy [21]. In the person-based evaluations, all studies, except the study by Ogdie et al [14], assessed multiple predetermined joints or locations irrespective of clinical manifestations using ultrasound to make the final diagnosis.

Ultrasound findings of gout include the double contour sign, presence of tophi, and the snowstorm sign which are reported to be specific for gout [16]. The presence of these three features is closely related to the deposition of monosodium urate crystals and is rarely seen in other types of arthritis [9]. This is consistent with the findings of this study, which showed high specificities in the diagnosis of gout using these three features in the person-based evaluations (all exceeded 0.90). However, the relatively modest or even low sensitivities (0.66, 0.56 and 0.31 for the DCS, presence of tophi and the snowstorm sign, respectively) imply that the absence of a single feature does not rule out the possibility of gout. By simultaneous consideration of these ultrasound characteristics, the overall pooled sensitivity of US for the diagnosis of gout in person-based evaluations was significantly improved. However, it remains unknown which and how many joints must be scanned by ultrasound to strike a balance between diagnostic accuracy, efficiency, and economic costs.

Data from the joint-/location-based evaluations were available to assess the diagnostic utility of the DCS and presence of tophi for gout. According to the literature, the knee and first metatarsophalangeal (MTP) joints are the most frequently affected joints in gout [11, 13, 17, 21, 25, 26]. Interestingly, this study found that, for the utility of the DCS in the diagnosis of gout, the sensitivity in joint-/location-based evaluations tends to be higher (but not significantly different) than that of the person-based evaluations. Meanwhile, the specificity in the joint-/location-based evaluations was significantly lower than that of the person-based evaluations. This observation may be explained by the fact that the investigators were apt to perform person-based evaluations for chronic and subacute gout patients who were more likely to have false negative findings of predetermined joints/locations on musculoskeletal ultrasound. On the other hand, the joint-/location-based evaluations included a substantial proportion of CPPD patients in the cross-sectional studies, which may have led to a decrease in the observed specificity.

False negative cases occur for various reasons. The presence of tophi, the DCS and the snowstorm signs are directly associated with the level of serum urate acid (SUA) [27, 28]. Das et al found that the DCS disappeared when the SUA level was maintained below 6 mg/dl for more than 6 months while dissolution of tophi took longer [15]. Meanwhile, multiple studies have demonstrated that these ultrasonographic signs appear in a specific sequential order. Normally, tophi develop late and the median duration of disease is 12.5 years in patients with tophi [14, 16, 27], which may explain the trend of a lower (but not significantly different) sensitivity and a similar specificity in the joint-/location-based evaluations compared to that in the person-based evaluations. In contrast, Elsaman et al found that, for well-established gout patients, the median disease duration was 2 years for those with the snowstorm sign on US and 5.5 years for those without, indicating that this early ultrasound feature has lower diagnostic value in a patient with a long history of gout [16]. Median disease duration for patients with the DCS on US was between those with the snowstorm sign and those with tophi [16]. The various studies analyzed in this systematic review included patient populations with different disease durations; this may partially explain the heterogeneous results among different studies.

There are some limitations to the current study. First, although 13 studies were included in the meta-analysis, sub-group analyses were conducted based on a small number of studies. Second, we included both cross-sectional studies and case-control studies. The quality of the primary studies greatly influences the quality of the meta-analysis. Some cross-sectional studies enrolled patients with inflamed joints without clear diagnoses, which represented those mostly likely to accept ultrasound examinations in clinical practice and gain benefits; however, other studies were conducted in established (somewhat advanced) gout patients in rheumatology clinics where the diagnosis was clear. In addition, the definitions of the control groups were different among the case-control studies included in this meta-analysis; some studies included control patients with inactive joint diseases that were unlikely to be gout, while others included healthy participants [15, 22]. Therefore, selection bias was inevitable. Third, the qualification of the sonographers, the device used, duration of symptoms, the ultrasound features taken into overall consideration, interpretation of ultrasound images among sonographers, the number of examined joints in person-based evaluations and other methodological characteristics varied across studies. Therefore, future studies are needed to refine the study design and investigate the performance of ultrasound at specific sites and at specific time points in the disease course of gout. Furthermore, follow-up should be recommended to observe the longitudinal changes of ultrasound features along and their relationship with SUA levels.

## Conclusions

In summary, our comprehensive meta-analysis demonstrates that ultrasound is a useful tool in the diagnosis of gout. The double contour sign, the presence of tophi and the snowstorm sign on ultrasound have high specificity but modest to low sensitivity in the diagnosis of gout for person-based evaluations. Simultaneously considering these features may improve the diagnostic sensitivity of ultrasound for gout. However, the double contour sign alone is weak in the differentiation of gout and non-gout for joint-/location-based evaluations. To warrant current conclusions, well-designed studies are still needed.

## Supporting information

S1 File PRISMA checklist. (DOC)

S2 File Editorial certificate. (PDF)

S1 Table Data of reported values for TP, FP, FN and TN for each study. (XLS)
